# Sirtuin 3 enhanced drug sensitivity of human hepatoma cells through glutathione S-transferase pi 1/JNK signaling pathway

**DOI:** 10.18632/oncotarget.10319

**Published:** 2016-06-29

**Authors:** Na-Na Tao, Hong-Zhong Zhou, Hua Tang, Xue-Fei Cai, Wen-Lu Zhang, Ji-Hua Ren, Li Zhou, Xiang Chen, Ke Chen, Wan-Yu Li, Bo Liu, Qiu-Xia Yang, Sheng-Tao Cheng, Li-Xia Huang, Ai-Long Huang, Juan Chen

**Affiliations:** ^1^ Key Laboratory of Molecular Biology for Infectious Diseases (Ministry of Education), Institute for Viral Hepatitis, Department of Infectious Diseases, The Second Affiliated Hospital, Chongqing Medical University, Chongqing, China; ^2^ Collaborative Innovation Center for Diagnosis and Treatment of Infectious Diseases, Zhejiang University, Zhejiang, China; ^3^ Department of Epidemiology, School of Public Health and Management, Chongqing Medical University, Chongqing, China

**Keywords:** hepatocellular carcinoma, SIRT3, drug sensitivity, glutathione S-transferase pi 1, c-Jun N-terminal kinase

## Abstract

SIRT3, a class III histone deacetylase, has been implicated in various cancers as a novel therapeutic target. In hepatocellular carcinoma (HCC), we previously reported that SIRT3 induced cell apoptosis by regulating GSK-3β/Bax signaling pathway. Downregulation of SIRT3 in HCC cells facilitates tumor cell survival. In this study, we found that chemotherapeutic agents (doxorubicin, cisplatin and epirubicin) and sorafenib treatment downregulated SIRT3 mRNA and protein levels in three HCC cell lines. MTS assay found that SIRT3 overexpression sensitized liver cancer cells to chemotherapeutic agents and sorafenib in SMMC-7721, Huh-7 and PLC/PRF/5 cell lines. Moreover, SIRT3 overexpression promoted chemotherapeutic agents-induced or sorafenib-induced apoptosis as evidenced by flow cytometry, enhanced PARP cleavage and enhanced Caspase-9 cleavage in three HCC cells. In contrast, SIRT3 silencing increased drug resistance of HCC cells to chemotherapeutic agents. Mechanistic study found that SIRT3 downregulated the mRNA and protein levels of glutathione S-transferase pi 1 (GSTP1), which is a member of phase II detoxification enzymes families involved in metabolizing for chemotherapeutic agents. Moreover, SIRT3 decreased the amount of GSTP1 that was associated with JNK, which finally contributed the activation of JNK activity and activation of downstream target c-Jun and Bim. Importantly, GSTP1 overexpression or JNK inhibitor abolished SIRT3-induced apoptosis in HCC cells exposed to chemotherapeutic agents. Finally, there was a negative correlation between SIRT3 expression and GSTP1 expression in human HCC tissues. Together, our findings revealed SIRT3 could enhance the drug sensitivity of HCC cells to an array of chemotherapeutic agents. SIRT3 may serve as a potential target for improving the chemosensitivity of HCC patients.

## INTRODUCTION

Hepatocellular carcinoma (HCC) is the fifth most frequently occurring cancer worldwide, with over 782,000 new cases estimated to have occurred in 2012 [1]. There are marked geographic variations in the distribution of HCC, with most cases occurring in Eastern Asia and sub-Saharan Africa [2]. China is one of the countries with a high incidence rate of HCC. Current therapeutic approaches for HCC include surgery, radiotherapy and chemotherapy. Although curative surgery is the most effective treatment, systemic chemotherapy on postoperative HCC patients has also been carried out to avoid recurrence and metastases. Doxorubicin, epirubicin, cisplatin, Folfox scheme (oxaliplatin, leucovorin and infusional fluorouracil (5-FU)) and sorafenib are so far the commonly used chemotherapeutic agents. Unfortunately, chemotherapeutic agents are modestly effective on HCC and drug resistance is the major obstacle in the therapy. Therefore, understanding of underlying mechanism of drug resistance is urgently needed to identify novel treatment.

Sirtuins, the class III histone deacetylases (HDACs), are nicotinamide adenine dinucleotide oxidized form (NAD^+^)-dependent deacetylases that target histone and nonhistone protein [3, 4]. Mammalian sirtuins (SIRT1 to SIRT7) has been implicated in a wide range of biological processes such as metabolism, senescence, apoptosis, DNA repair and carcinogenesis [5–10]. Among these seven members, SIRT3 is generally believed to be a mitochondrial deacetylase that functions as a regulator of mitochondrial metabolic and oxidative stress regulatory pathways. The loss of SIRT3 mitochondrial function can cause cell damage, and provide an environment permissive to carcinogenesis [11, 12]. Increasing evidences have linked SIRT3 in tumor development. Aberrant expression of SIRT3 is detected in breast cancer, oral cancer, squamous cell carcinoma, melanoma, and hepatocellular carcinoma [13–17].

We previously reported that SIRT3 was frequently downregulated in HCC tissues and cell lines. Ectopic expression of SIRT3 induced cell apoptosis via the GSK-3β/Bax signaling pathway in HCC cells [17]. In this study, we found treatment of chemotherapeutic agents could inhibit SIRT3 expression in serval HCC cell lines. Ectopic expression SIRT3 sensitized HCC cells to treatment of chemotherapeutic agents by modulating GSTP1/JNK signaling pathway. Our data suggest a role for SIRT3 in chemosensitivity of HCC cells.

## RESULTS

### Chemotherapeutic agents inhibited SIRT3 expression in HCC cells

**Figure 1:**
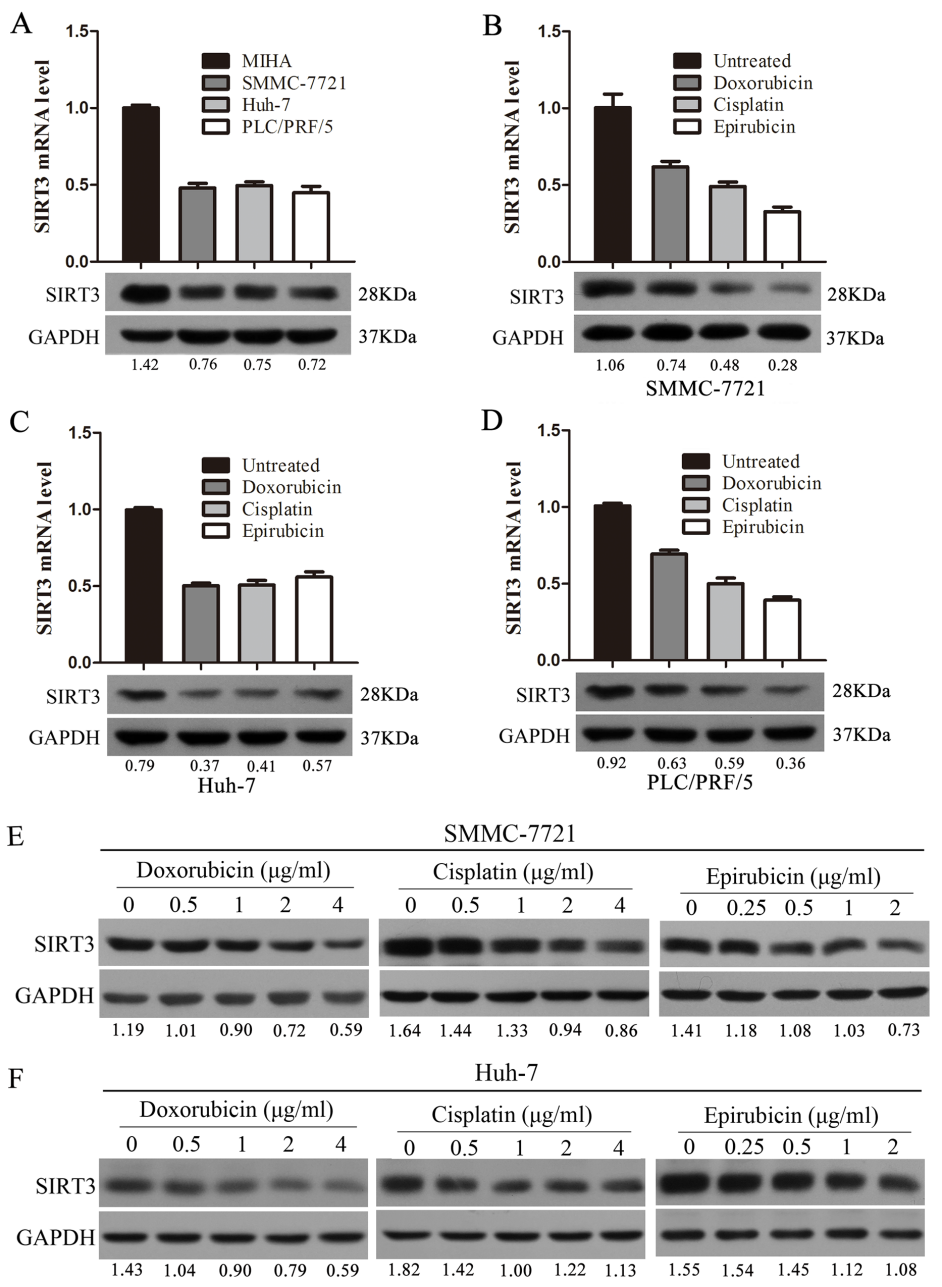
Chemotherapeutic agents inhibited SIRT3 expression in HCC cells **A.** SIRT3 expression in three HCC cell lines (SMMC-7721, Huh-7 and PLC/PRF/5) and one immortalized liver cell line (MIHA) by using qPCR and western blotting analysis. β-actin mRNA expression was used as an internal control for qPCR. GAPDH was used as a loading control in western blotting analysis. **B-D.** Chemotherapeutic agents (doxorubicin, cisplatin and epirubicin) inhibited SIRT3 expression. SMMC-7721(B), Huh-7(C) and PLC/PRF/5 (D) cells were treated with doxorubicin (1 μg/ml), cisplatin (1 μg/ml) or epirubicin (0.5 μg/ml) for 48 h before subjected to qPCR and western blotting analysis. **E-F.** The expression of SIRT3 was examined in SMMC-7721 and Huh-7 cells treated with various concentrations of chemotherapeutic agents (doxorubicin, cisplatin and epirubicin) by using western blotting analysis. GAPDH was used as a loading control.

We first examined the expression of SIRT3 in three HCC cell lines (SMMC-7721, Huh-7 and PLC/PRF/5) and one immortalized liver cell line (MIHA) by using qPCR and western blotting analysis. The results showed that both mRNA and protein levels of SIRT3 in HCC cell lines were markedly lower relative to MIHA cells, indicating that SIRT3 was frequently downregulated in HCC cells (Figure 1A).

To determine whether chemotherapeutic agents could alter SIRT3 expression, HCC cells were treated with doxorubicin, cisplatin and epirubicin. Treatment of chemotherapeutic agents significantly inhibited SIRT3 both mRNA and protein levels in SMMC-7721 cells (Figure 1B). Similar decreased SIRT3 expression in response to chemotherapeutic agents was also observed in Huh-7 and PLC/PRF/5 cells (Figure 1C–1D). Furthermore, the expression of SIRT3 was also examined in HCC cells which treated with a series of concentrations of chemotherapeutic agents. Western blotting analysis revealed that chemotherapeutic agents inhibited SIRT3 expression in HCC cells in a dose-dependent manner (Figure 1E–1F). These data suggested that SIRT3 may play a role in the drug sensitivity of HCC cells.

### SIRT3 overexpression sensitized HCC cells to chemotherapeutic agents

**Figure 2:**
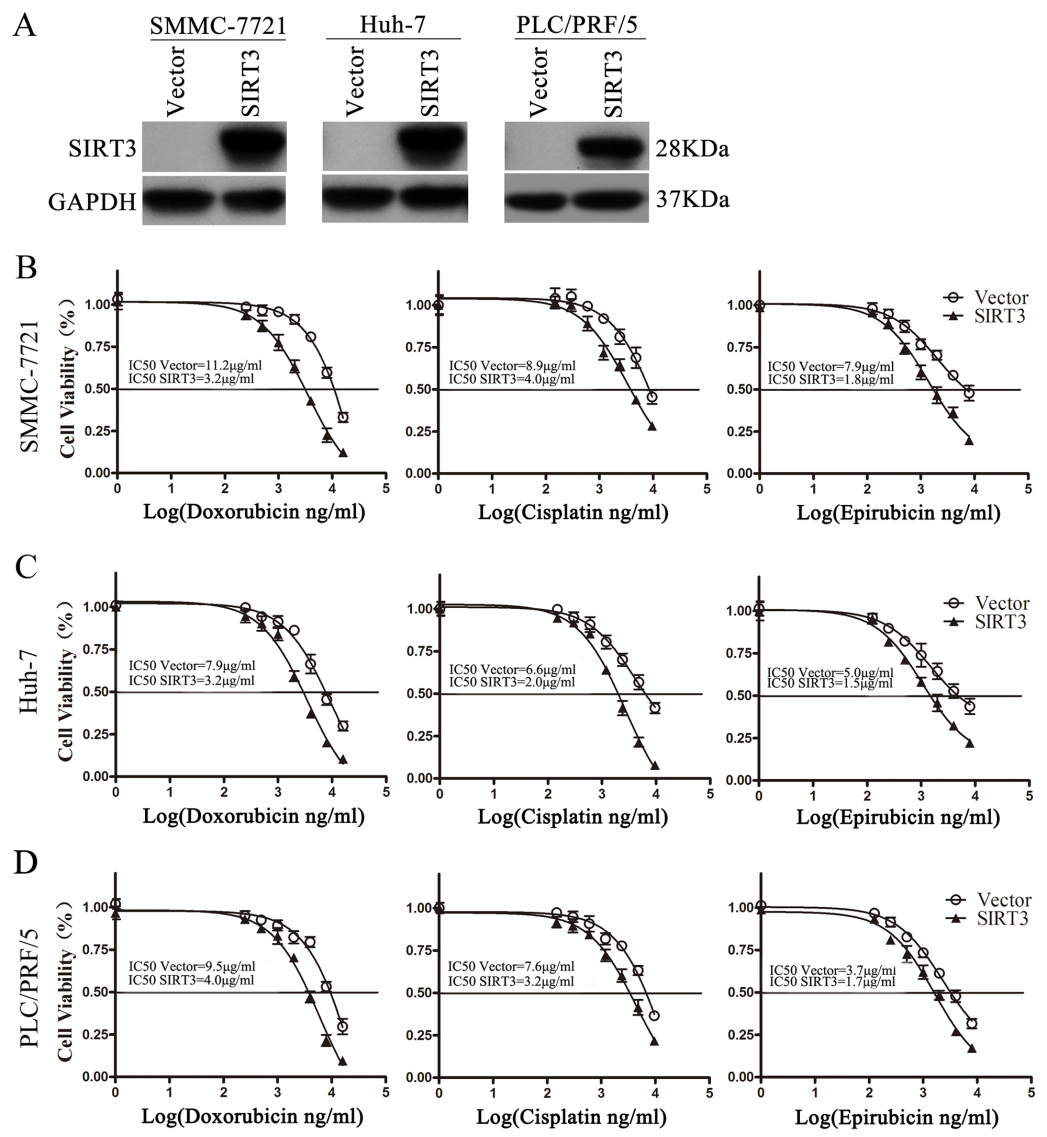
SIRT3 overexpression sensitized HCC cells to chemotherapeutic agents **A.** Western blotting analysis of SIRT3 proteins in cells infected with lentivirus expressing SIRT3 or vector. GAPDH was used as a loading control. **B-D.** Cell viability of SIRT3-overexpressing cells exposed to chemotherapeutic agents. Forty-eight hour after lentivirus infection, SMMC-7721(B), Huh-7(C) and PLC/PRF/5 (D) were treated with various concentrations of doxorubicin, cisplatin or epirubicin for 48 h. Cell viability was determined by MTS assay.

To examine the potential role of SIRT3 in resistance to anticancer therapies of HCC, HCC cells were infected with lentivirus expressing SIRT3 and then exposed to various concentrations of doxorubicin, cisplatin and epirubicin. SIRT3 was overexpressed markedly in all three HCC cell lines examined (Figure 2A). MTS assay found that SIRT3 overexpression significantly enhanced cellular susceptibility to three chemotherapeutic agents in SMMC-7721, Huh-7 and PLC/PRF/5 cells (Figure 2B–2D).

**Figure 3:**
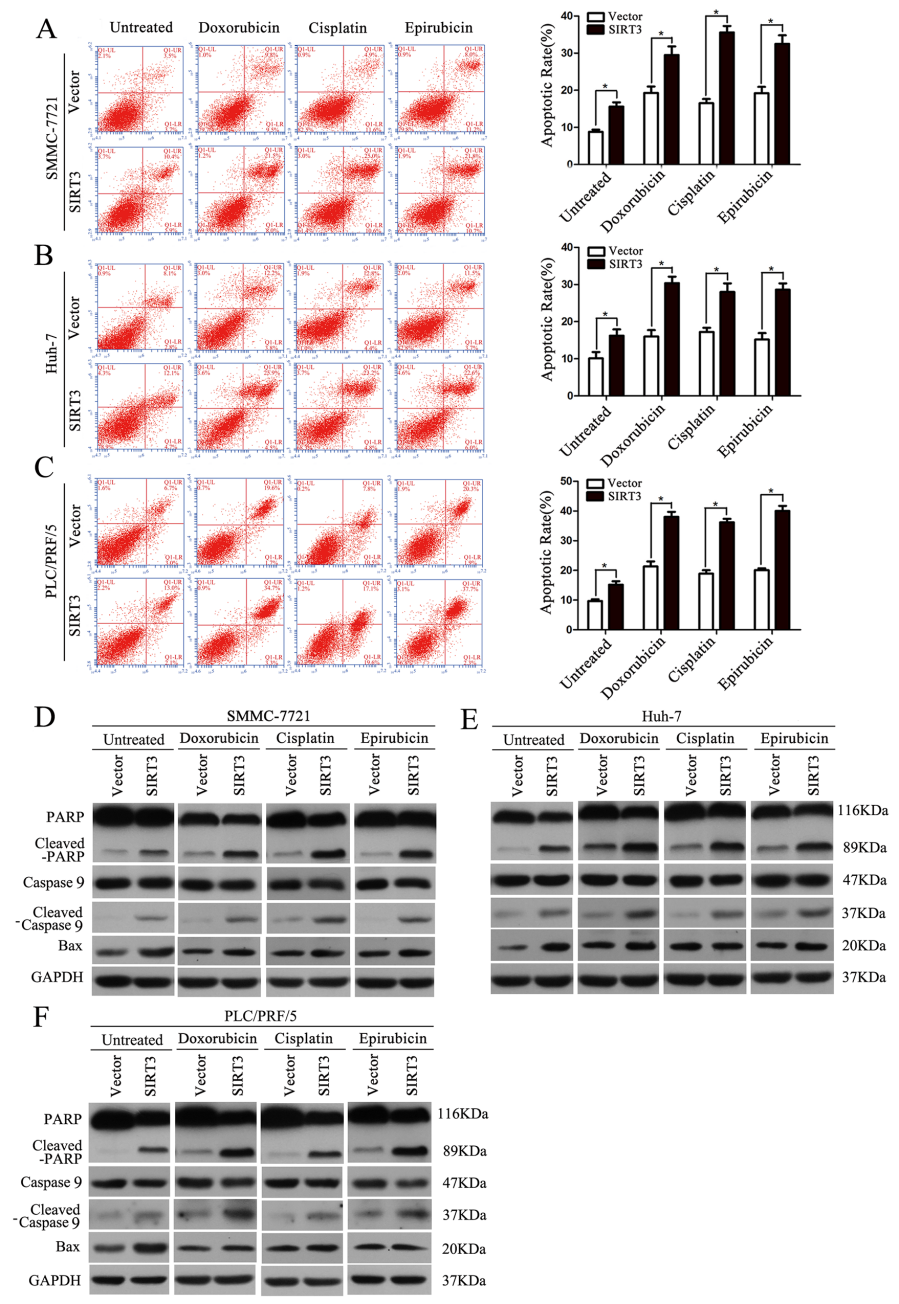
SIRT3 overexpression enhanced chemotherapeutic agents-induced apoptosis in HCC cells **A-C.** Apoptosis in different groups was analyzed by flow cytometry with Annexin V/PI. Forty-eight hour after lentivirus infection, SMMC-7721, Huh-7 and PLC/PRF/5 cells were treated with doxorubicin (1 μg/ml), cisplatin (1 μg/ml) or epirubicin (0.5 μg/ml) for 48 h and subjected to flow cytometry analysis. *, *P*<0.05. All histograms show mean values from 3 independent experiments. **D-F.** PARP cleavage, caspase 9 cleavage and Bax expression in different groups were analyzed by western blotting analysis. GAPDH was used as a loading control.

The sensitizing effect of SIRT3 of HCC cells was further evidenced by flow cytometry and western blotting analysis of several apoptotic markers. Although SIRT3 overexpression induced increased apoptosis rate in untreated HCC cells, chemotherapeutic agents treatment in SIRT3-overexpressing HCC cells resulted in a much more increase in the apoptosis rate compared to their respective control cells (Figure 3A–3C). Furthermore, several apoptotic markers including cleaved-PARP and cleaved-caspase 9 were analyzed. Concordantly, SIRT3 overexpression also increased expression of cleaved-PARP, cleaved-caspase 9 in untreated HCC cells. However, SIRT3 overexpression resulted in more increased level of cleaved-PARP, cleaved-caspase 9 in chemotherapeutic agents-treated HCC cells (Figure 3D–3F). We previously reported that SIRT3 induces apoptosis in HCC cells via activating Bax signaling [17]. In this study, we also examine the effect of SIRT3 on Bax activation. SIRT3 overexpressing indeed induced the expression of Bax in untreated HCC cells. However, SIRT3 had no effect on Bax expression in chemotherapeutic agents-treated HCC cells (Figure 3D–3F). Together, these data suggested that SIRT3 may play a role in the regulation of drug sensitivity in HCC cells independent on Bax-mediated signaling.

### SIRT3 knockdown in HCC cells induced drug resistance against chemotherapeutic agents

To further elucidate the role of SIRT3 in drug sensitivity of HCC cells, HCC cells (SMMC-7721 and Huh-7) were transfected with siRNA targeting SIRT3 and then exposed to various concentrations of doxorubicin, cisplatin and epirubicin. The silence of SIRT3 was measured by western blotting (Supplementary Figure S1A). SIRT3 knockdown significantly increased drug resistance to chemotherapeutic agents examined in both SMMC-7721 and Huh-7 cells (Supplementary Figure S1B). Furthermore, AnnexinV/PI assays indicated a dramatic reduction in the apoptosis rate in SIRT3-depleted cells compared to their respective controls in response to chemotherapeutic agents (Supplementary Figure S1C).

### SIRT3 overexpression sensitized HCC cells to Sorafenib

**Figure 4:**
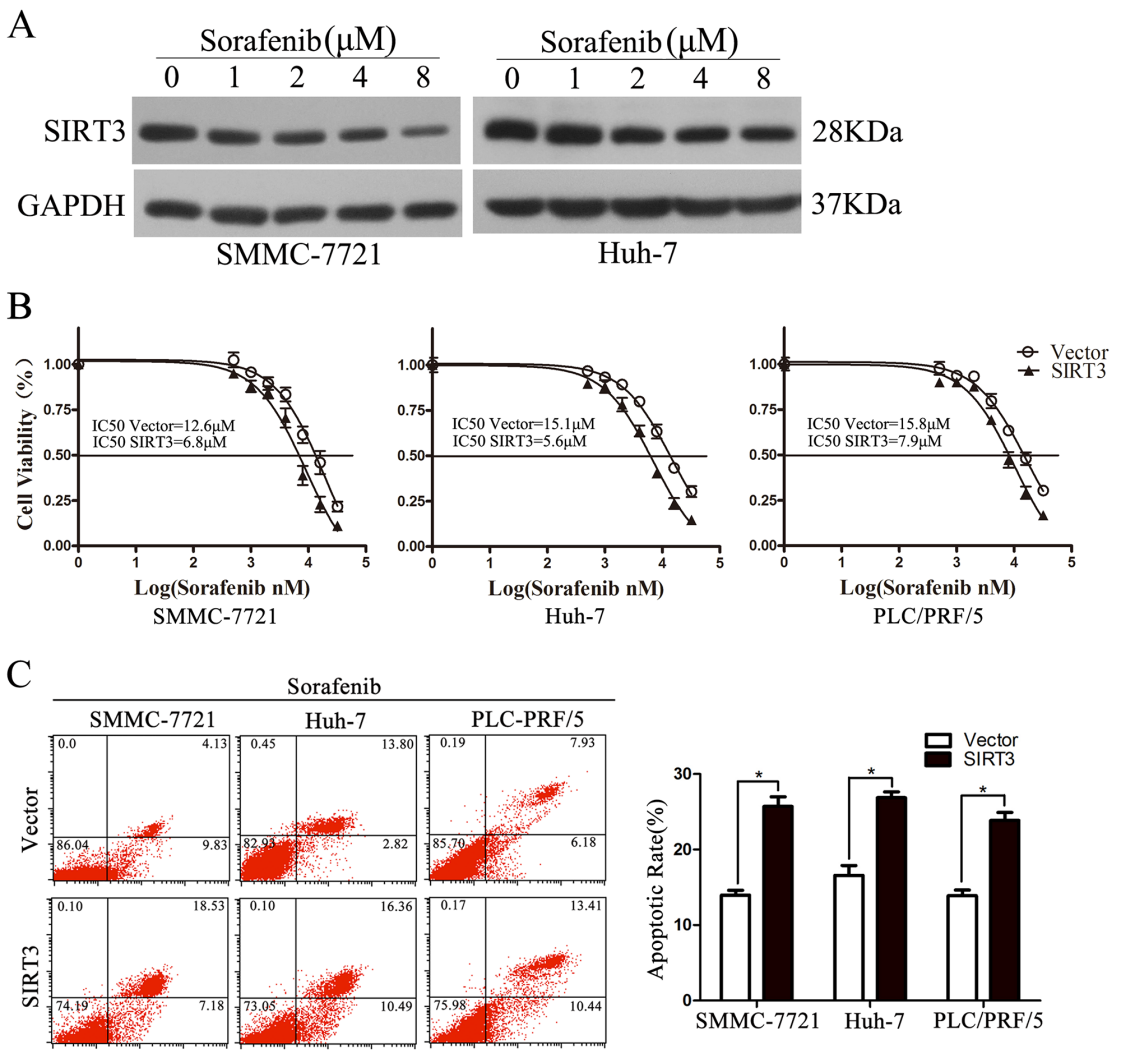
SIRT3 overexpression sensitized HCC cells to Sorafenib **A.** Sorafenib inhibited SIRT3 expression in HCC cells. The expression of SIRT3 was examined in SMMC-7721 and Huh-7 cells treated with various concentrations of sorafenib. **B.** Cell viability of SIRT3-overexpressing cells exposed to Sorafenib (2 μM). Forty-eight hour after lentivirus infection, SMMC-7721, Huh-7 and PLC/PRF/5 cells were treated with various concentrations of Sorafenib and then subjected to MTS assay. **C.** SIRT3 enhanced Sorafenib-induced apoptosis. Forty-eight hour after lentivirus infection, SMMC-7721, Huh-7 and PLC/PRF/5 cells were treated with Sorafenib (2 μM) for 48 h before flow cytometry with Annexin V/PI. *, *p*<0.01. All histograms show mean values from 3 independent experiments.

Sorafenib, a multiple kinase inhibitor, is the first and only drug that is clinically approved for patients with advanced HCC [18]. We further examined the potential role of SIRT3 in resistance to the sorafenib in SMMC-7721, Huh-7 and PLC/PRF/5 cells. The expression of SIRT3 was examined in SMMC-7721 and Huh-7 cells treated with various concentrations of sorafenib. Western blotting analysis revealed that sorafenib inhibited SIRT3 expression in HCC cells in a dose-dependent manner (Figure 4A). MTS assay found that SIRT3 overexpression significantly sensitized HCC cells to sorafenib treatment (Figure 4B). The sensitizing effect was further examined by an increase of cell apoptosis rate in response to sorafenib treatment in all three HCC cell lines as evidenced by flow cytometry (Figure 4C). Together, these data suggested that targeting SIRT3 sensitizes HCC cells to oncogenic kinase inhibitor.

### SIRT3 overexpression regulated GSTP1/JNK signaling pathway

**Figure 5:**
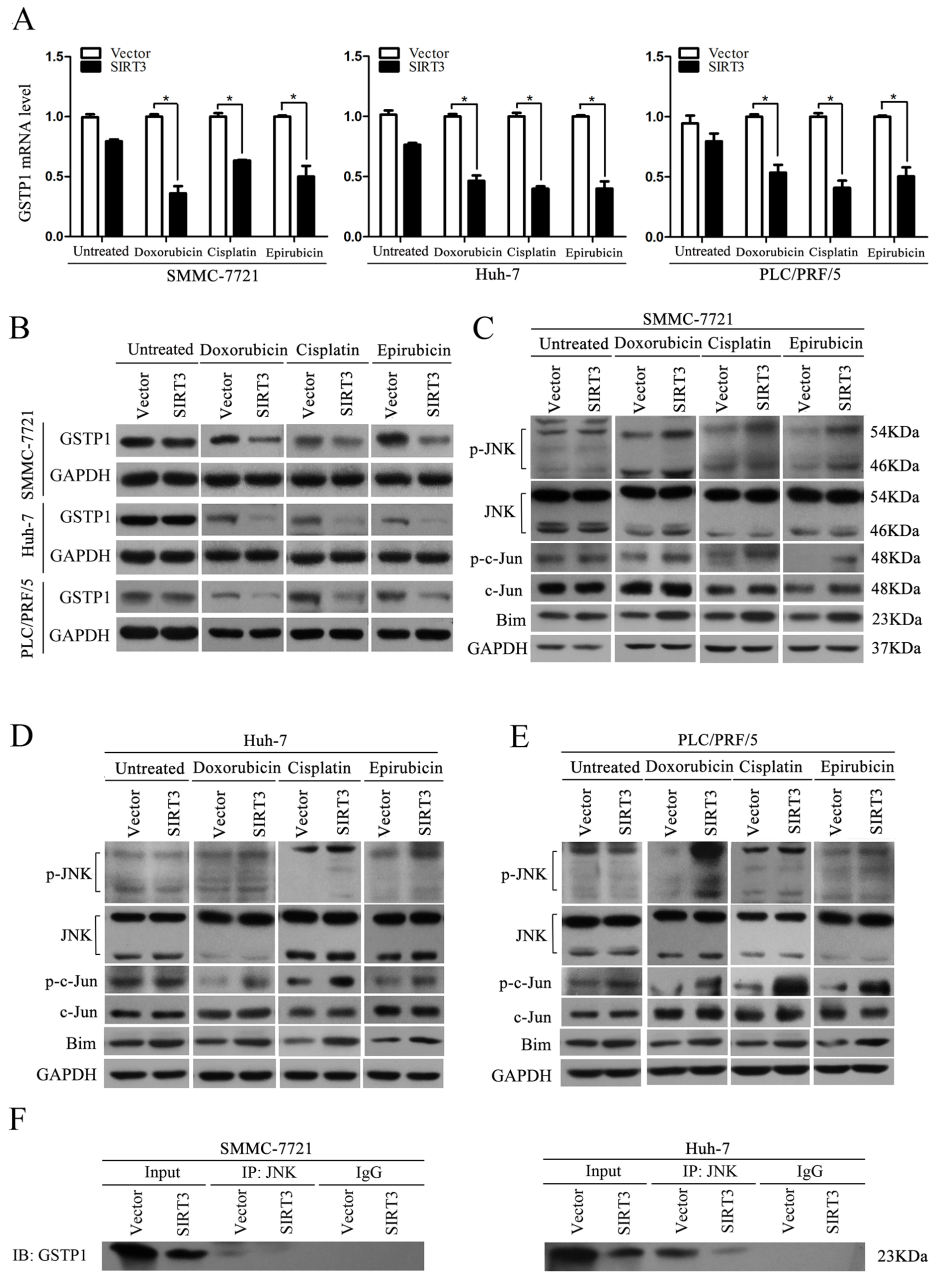
SIRT3 overexpression regulated GSTP1/JNK signaling pathway **A.** SIRT3 inhibited GSTP1 mRNA level. Forty-eight hour after lentivirus infection, SMMC-7721, Huh-7 and PLC/PRF/5 cells were treated with doxorubicin (1 μg/ml), cisplatin (1 μg/ml) or epirubicin (0.5 μg/ml) for 48 h. The mRNA level of GSTP1 was detected by qPCR. β-actin mRNA expression was used as an internal control. **B.** SIRT3 inhibited GSTP1 protein level. The protein level of GSTP1 in SMMC-7721, Huh-7 and PLC/PRF/5 cells with or without chemotherapeutic agents was determined by western blot analysis. GAPDH was used as a loading control. **C-E.** The expression of total JNK, total c-Jun, phosphorylated-JNK, phosphorylated-c-Jun and Bim were examined in SMMC-7721 (C), Huh-7 (D) and PLC/PRF/5 (E) cells treated with doxorubicin (1 μg/ml), cisplatin (1 μg/ml) or epirubicin (0.5 μg/ml). GAPDH was used as a loading control. **F.** SIRT3 decreased the amount of GSTP1 that was associated with JNK. Immunoprecipitation was conducted with lysates prepared from SIRT3-overexpressing HCC cells (SMMC-7721 and Huh-7) or control cells treated with doxorubicin (1 μg/ml) by anti-JNK antibody, and immunoblotting was performed with anti-GSTP1 antibody.

Glutathione S-transferases (GSTs) are a family of phase II detoxification enzymes that catalyze the conjugation of glutathione (GSH) to a wide variety of electrophilic compounds [19]. Glutathione S-transferase pi 1 (GSTP1) is the most studied one of GSTs family which implicated in cellular resistance. To investigate the underlying mechanism of chemosensitizing effect of SIRT3, we examined the expression of GSTP1 in SIRT3-overexpressing cells under treatment of chemotherapeutic agents. QPCR results showed that SIRT3 overexpression inhibited the mRNA level of GSTP1 in the three HCC cells under treatment of chemotherapeutic agents (Figure 5A). Moreover, ectopic expression of SIRT3 inhibited protein level of GSTP1 in HCC cells treated with chemotherapeutic agents (Figure 5B). However, SIRT3 overexpression has no effect on GSTP1 expression in untreated control cells. To exclude the possibility that chemotherapeutic agents have effect on GSTP1 expression, we detected mRNA and protein levels of GSTP1 in HCC cells treated with chemotherapeutic agents. Both qPCR and western blotting analysis found that the chemotherapeutic agents had no effect on GSTP1 expression (Supplementary Figure S2). Overall, these data suggested that GSTP1 may be involved in the drug sensitivity mediated by SIRT3 in HCC cells.

GSTP1 has been shown to associate with c-Jun N-terminal kinase (JNK) pathways involved in cell survival and death signaling. The interaction between GSTP1 and JNK inhibits the kinase activity of JNK, which finally results in the inactivation of c-Jun, a component of the activator protein-1 (AP-1) transcription factor. The activation of AP-1 leads to induction of AP-1-dependent target genes involved in cell death [20]. Therefore, we further analyzed JNK signaling pathway in SIRT3-overexpressing cells. SIRT3 overexpression promoted the phosphorylation status of JNK whereas it had no effect on total JNK expression in all three HCC cells (SMMC-7721, Huh-7 and PLC/PRF/5) treated with chemotherapeutic agents (Figure 5C–5E). Moreover, SIRT3 overexpression resulted in increased level of phosphorylated-c-Jun and Bim which is downstream target of AP-1 in HCC cells exposed to chemotherapeutic agents (Figure 5C–5E). However, SIRT3 also did not change the expression of JNK, p-JNK, c- Jun, p-c-Jun and Bim in untreated control cells. We further examined whether SIRT3 could affect the association between GSTP1 and JNK by using co-immunoprecipitation assay. Our data showed that SIRT3 overexpression decreased the amount of GSTP1 that was associated with JNK (Figure 5F). Taken together, these data suggested that under chemotherapeutic agents treatment, SIRT3 inhibited GSTP1 expression, which resulted in decreased GSTP1 interacting with JNK. The interaction of the GSTP1: JNK complex dissociated in SIRT3-overexpressing HCC cells finally contributed the activation of JNK activity and activation of downstream target c-Jun.

### GSTP1 overexpression or JNK inhibitor attenuate the sensitizing effect of SIRT3

**Figure 6:**
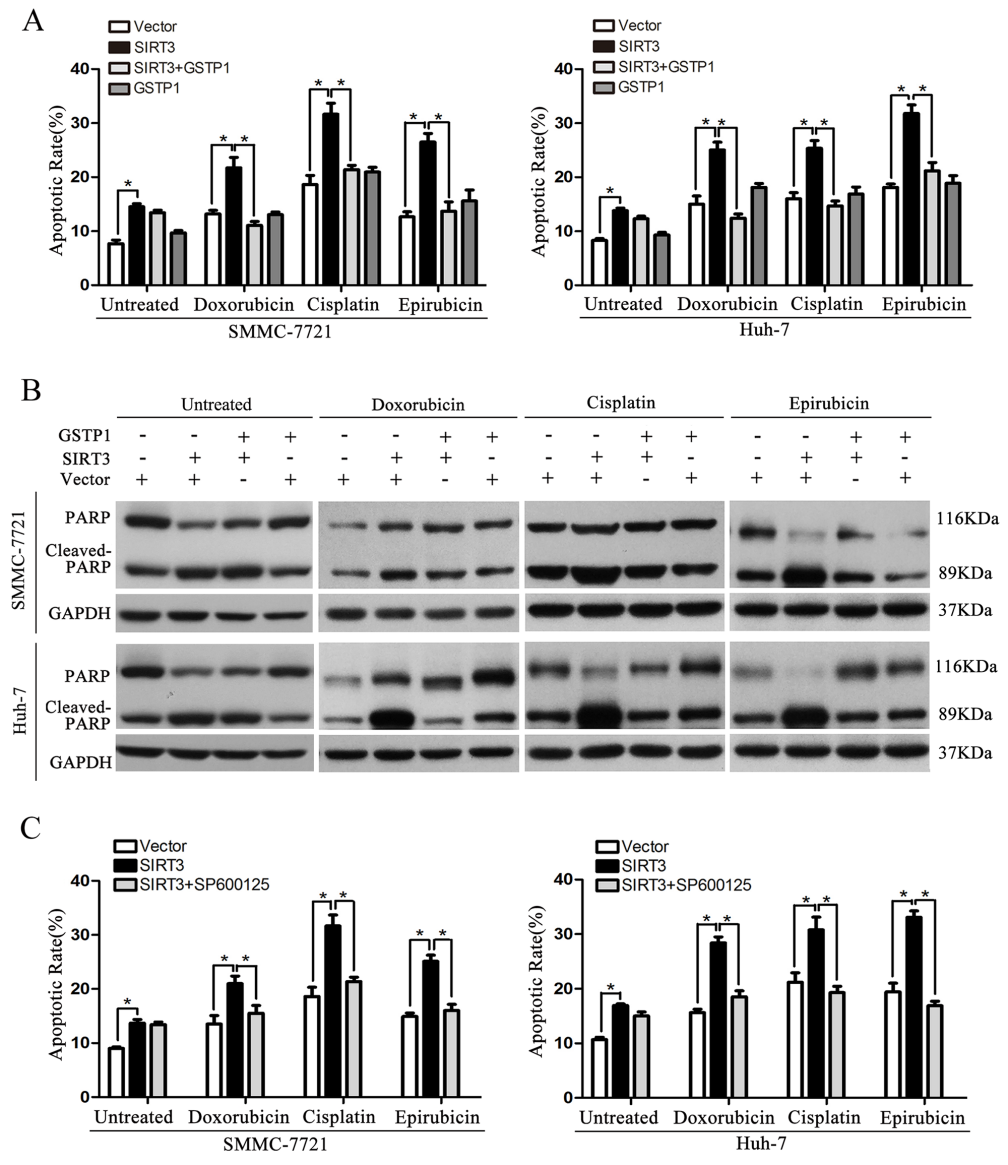
GSTP1 overexpression or JNK inhibitor attenuate the sensitizing effect of SIRT3 **A-B.** GSTP1 overexpression abolished SIRT3-induced apoptosis in HCC cells treated with chemotherapeutic agents. SMMC-7721 and Huh-7 cells stably expressing SIRT3 were transfected with plasmid expressing GSTP1 and were then exposed to doxorubicin (1 μg/ml), cisplatin (1 μg/ml) or epirubicin (0.5 μg/ml) for 48 h. Apoptotic ratio in different groups was detected by flow cytometry with Annexin V/PI(A) and PARP cleavage analysis (B). *, *p*<0.05. **C.** JNK inhibitor abolished SIRT3-induced apoptosis in HCC cells treated with chemotherapeutic agents. SMMC-7721 and Huh-7 cells stably expressing SIRT3 were treated with JNK inhibitor SP600125 (10 μM) and chemotherapeutic agents. Apoptotic ratio in different groups was detected by flow cytometry. *, *p*<0.05.

To further determine whether SIRT3 exerts its function by modulating GSTP1 signaling pathway, we overexpressed GSTP1 in SMMC-7721 and Huh-7 cells stably expressing SIRT3. The apoptosis ratios of HCC cells were detected by both flow cytometry and PARP cleavage. Importantly, these cells treated with chemotherapeutic agents showed a significant reduction in apoptosis rate compared with cells ectopically expressing SIRT3 without GSTP1 overexpression (Figure 6A and Supplementary Figure S3A). Consistently, PARP cleavage analysis confirmed that GSTP1 expression abolished SIRT3-induced apoptosis in HCC cells exposed to doxorubicin, cisplatin and epirubicin (Figure 6B). However, GSTP1 overexpression in untreated HCC cells had no effect on SIRT3-induced apoptosis (Figure 6A–6B). We also treated HCC cells expressing SIRT3 with SP600125, a specific JNK inhibitor. Flow cytometry revealed that treatment of SP600125 had no effect on SIRT3-induced apoptosis in untreated HCC cells while it abolished cell apoptosis induced by SIRT3 in HCC cells exposed to chemotherapeutic agents (Figure 6C and Supplementary Figure S3B). Altogether, these data suggested SIRT3 could increase drug sensitivity of HCC cells to anticancer agents via GSTP1 signaling pathway.

### The correlation between SIRT3 and GSTP1 in HCC tissues

**Figure 7:**
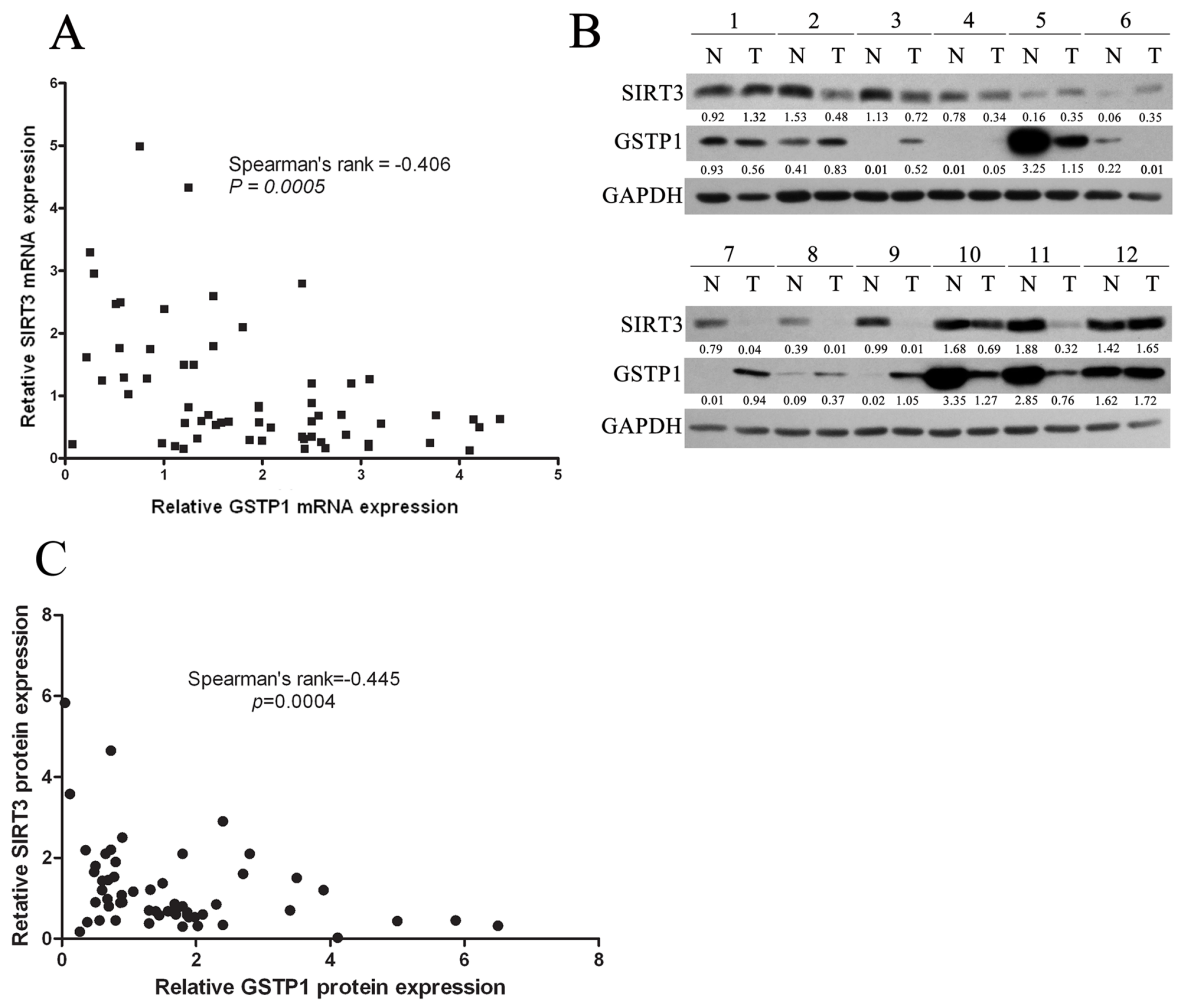
The correlation between SIRT3 and GSTP1 in 60 paired HCC tissues **A.** SIRT3 mRNA negatively correlated with GSTP1 mRNA in 60 paired HCC tissues. SIRT3 and GSTP1 mRNA was first normalized by the expression level of β-actin, and the induction of SIRT3 and GSTP1 in HCC over nontumoral liver in each patients was calculated. Correlation analysis was analyzed by Spearman's σ rank test. **B.** Representative western blotting of 12 paired HCC tissues showing SIRT3 and GSTP1 expression. **C.** SIRT3 protein negatively correlated with GSTP1 protein in 60 paired HCC tissues. SIRT3 and GSTP1 protein was first normalized by the expression level of β-actin, and the induction of SIRT3 and GSTP1 in HCC over nontumoral liver in each patient was calculated. Correlation analysis was analyzed by Spearman's σ rank test.

Finally, we analyzed the relevance of SIRT3 and GSTP1 expression in 60 paired HCC tissues by using qPCR and western blotting analysis. Correlative analysis revealed a negative correlation between SIRT3 mRNA and GSTP1 mRNA levels (Spearman's rank=−0.406, *p*=0.0005) (Figure 7A). Moreover, SIRT3 protein level negatively correlated with GSTP1 protein level (Spearman's rank=-0.445, *p*=0.0004) (Figure 7B–7C). These data suggested that the SIRT3-GSTP1 regulatory axis might exist *in vivo*.

## DISCUSSION

SIRT3, the primary mitochondrial deacetylase in sirtuins family, regulates metabolism, ATP generation, aging, cell survival and death, and carcinogenesis [11, 21]. The role of SIRT3 in carcinogenesis is controversial. Allison and colleagues found that SIRT3 is required for apoptosis induced by selectively silencing Bcl-2 in HCT116 human epithelial cancer cells [22]. Also, Schumacker et al demonstrated SIRT3 suppresses the proliferation and survival of tumor cells through its effect on inhibits ROS production, as well as HIF-1a stabilization and its downstream transcriptional activity, thereby decreasing tumorigenesis [23]. On the contrary, SIRT3 is overexpressed in oral squamous cell carcinoma (OSCC), and down-regulation of it inhibited OSCC cell growth and proliferation in vitro and in vivo [14]. High expression of SIRT3 is associated with shorter survival time in esophageal cancer patients [24]. Therefore, the role of SIRT3 in neoplasia is cell type-specific, and potentially quite complex. Recently, emerging evidences have implicated the function of SIRT3 in drug resistance. Kaempferol treatment increased the expression and the mitochondria localization of the SIRT3 in chronic myelogenous leukemia cell K562. K562 cells stably overexpressing SIRT3 were more sensitive to kaempferol treatment [25]. SIRT3 altered sensitivity of breast cancer cells to tamoxifen (Tam), a commonly used anti-estrogen agent. SIRT3 was rapidly up-regulated in the Tam-sensitive MCF-7 cells following exposure to Tam. SIRT3 overexpression in MCF-7 cells decreased cellular sensitivity to Tam and blocked the Tam-induced apoptosis [26]. Moreover, SIRT3 protects cardiomyocytes from doxorubicin-induced oxidative damage by attenuating DOX-induced ROS production and rescuing mitochondrial respiration [27]. In HCC cells, we previously reported SIRT3 was frequently downregulated in HCC tissues and cell lines. SIRT3 overexpression significantly induced cell apoptosis in several HCC cell lines [17]. However, the role of SIRT3 in drug sensitivity of liver cancer cells remains elusive. In this study, we first found that chemotherapeutic agents (doxorubicin, cisplatin and epirubicin) and sorafenib treatment downregulated SIRT3 mRNA and protein levels in three HCC cell lines, which suggesting SIRT3 might play a regulatory role in drug sensitivity of HCC cells. MTS assay further confirmed that SIRT3 overexpression sensitized liver cancer cells to chemotherapeutic agents and sorafenib in SMMC-7721, Huh-7 and PLC/PRF/5 cell lines. Moreover, SIRT3 overexpression promoted chemotherapeutic agents-induced or sorafenib induced-apoptosis as evidenced by flow cytometry and enhanced PARP cleavage. Taken together, these results indicated the sensitizing effect of SIRT3 in chemotherapy in HCC. This finding may provide a possibility to combine SIRT3 activator together with chemotherapeutic agents as a novel treatment strategy for HCC. However, the results are still preliminary, and the synergistic role of SIRT3 activator in chemotherapy of HCC needs to be determined in animal models.

Mechanistic study of SIRT3-medicated chemosensitization was further investigated. We revealed that SIRT3 downregulated Glutathione S-transferase pi 1 (GSTP1) expression, which is a member of glutathione S-transferases (GSTs) family. GSTs belong to a superfamily of phase II detoxification enzymes that catalyze the conjugation of glutathione (GSH) to a wide variety endogenous and exogenous electrophilic compounds [28]. Human GSTs are divided into three families: cytosolic GSTs, mitochondrial GSTs and membrane-bound microsomal GSTs. GSTP1 is the most closely cytosolic GSTs that related to carcinogenesis and drug resistance [29]. GSTP1 overexpression have been reported in many cancers, such as breast cancer, colon cancer, kidney cancer, lung cancer, and ovarian cancer [20]. The sensitivity of these tumors toward chemotherapeutic agents, including cisplatin, doxorubicin and epirubicin, are negatively regulated by overexpression of GSTP1 [30–32]. In HCC, dysregulation of GSTP1 was observed in liver cancer cell lines [33], and in more than 77.8% of HBV-associated HCC tissues [34]. Chen and colleagues found that AG and GG genotypes of GSTP1 gene increased the risk of HCC [35]. Recently, a meta-analysis indicated a significant association between GSTP1 methylation and poor outcomes in HCC patients [36]. In this study, we found that SIRT3 decreased GSTP1 in both mRNA and protein levels which suggested SIRT3 regulated GSTP1, at least in part, occurs at a transcriptional level in liver cancer cells. This finding led us to hypothesize that SIRT3 may selectively controlled key factors involved in DNA methylation, histone modification and chromosome remodeling which affect GSTP1 gene transcription. In addition to GSH-conjugating activity, GSTP1 has been shown to associate with the c-Jun N-terminal kinases (JNK) pathways involved in cell survival and death signaling. GSTP1 functions to sequester the JNK kinase in a complex, thus preventing it from acting on downstream targets [20, 28]. In this study, we found that SIRT3 overexpression decreased the expression of GSTP1 associated with JNK, finally contributed the activation of JNK activity and activation of downstream target c-Jun. Importantly, GSTP1 overexpression or JNK inhibitor abolished SIRT3-induced apoptosis in HCC cells exposed to chemotherapeutic agents or sorafenib. Taken together, these data suggested that SIRT3 could sensitize HCC cells to chemotherapeutic agents via GSTP1/JNK signaling pathway.

In summary, we have identified a novel SIRT3/GSTP1/JNK pathway orchestrating cell death evasion and sensitivity to chemotherapy in HCC cells. In HCC cells, downregulation of SIRT3 facilitated cell death evasion and drug resistance to chemotherapy. Ectopic expression of SIRT3 not only induced cell apoptosis but also increased drug sensitivity to chemotherapeutic agents in HCC cells. Therefore, SIRT3 is a promising target in combination with chemotherapy or targeting therapies for HCC in future.

## MATERIALS AND METHODS

### Cell culture and transfection

Immortalized liver cell line (MIHA) and hepatocellular carcinoma cell lines (SMMC-7721, Huh-7 and PLC/PRF/5) were maintained in Dulbecco's modified Eagle's medium (DMEM) containing 10% fetal bovine serum (Gibco, USA), 100U/ml penicillin and 100ug/ml streptomycin (Thermo, USA). All cells were maintained in a humidified incubator at 37°C with 5% CO_2_. Transfection was carried out using X-tremeGENE HP DNA Transfection Reagent (Roche, Germany). Cells were authenticated by short tandem repeat (STR) fingerprinting by Beijing Microread Genetics Company Limited recently. SMMC-7721, Huh-7 and PLC/PRF/5 cells were treated with doxorubicin (1μg/ml), cisplatin (1μg/ml), epirubicin (0.5μg/ml), and sorafenib (2 μM) for 48 h after infection of lentivirus expressing SIRT3.

### Plasmids, antibodies and chemotherapeutic agents

The plenti6-SIRT3 expression vector was cloned as previously reported [17]. The pCMV6-XL5-GSTP1 expression vector (SC119655) was obtained from Origene (USA). Anti-SIRT3 (#2627), Anti-PARP (#9542), Anti-GSTP1 (#3369), Anti-c-Jun (#9165), Anti-phospho-c-Jun (#2361), Anti-phospho-JNK (#9251), Anti-Bim (#2933), Anti-Bax (#5023), Anti-Caspase 9(#9502) antibodies were obtained from Cell Signaling Technology (USA). Anti-GAPDH (sc-365062), Anti-JNK (sc-571) and Anti-IgG (sc-66931) antibodies were obtained from Santa Cruz Biotechnology (USA). Doxorubicin (D1515) and cisplatin (P4394) were obtained from Sigma-Aldrich (USA), epirubicin (56390-09-1) was obtained from Cayman Chemical (USA), Sorafenib (S7397) was obtained from Selleckchem (USA). JNK inhibitor SP600125 (S5567) was obtained from Sigma-Aldrich (USA).

### Cell proliferation assay

Cell proliferation in response to chemotherapeutic agents on cell was detected by MTS assay according to the manufacturer's instruction (Promega, USA).

### RNA extraction and quantitative real-time PCR (qPCR)

Total RNA was prepared using TRIzol reagent (Invitrogen, USA) and cDNA was synthesized from 1 μg of total RNA using the iScript® cDNA Synthesis Kit (Bio-Rad, USA). cDNA was analyzed by quantitative PCR using FastStart Universal SYBR Green Master Mix (Roche, Germany) on an IQTM 5 Multicolor Real-Time PCR Detection system (Bio-Rad, USA). β-actin was used as an endogenous control. The expression values of target genes were calculated using the 2^−ΔΔCt^ method. Values represent the mean ± SD of three independent experiments. The primer sequence: SIRT3 (F: CAAAGCTGGTTGAAGCTCAT, R: AAGGGTCTTTGGCAGACTGT), GSTP1 (F: GGGCA AGGATGACTATGTGA, R: AGCAGGTTGTAGTCAG CGAA).

### Western blotting analysis

Total proteins were extracted using RIPA lysis buffer containing EDTA-free protease inhibitor cocktail tablets (Roche, Germany) and the protein concentrations were determined using the BCA Protein Assay (Thermo, USA). Total proteins were resolved by sodium dodecyl sulfate-polyacrylamide gel electrophoresis (SDS-PAGE) and transferred to nitrocellulose membranes (GE Healthcare, UK). Western blotting analysis was performed with the indicated primary antibodies and then with horseradish peroxidase conjugated secondary antibodies. The blots were developed with immobilon western chemiluminescent HRP substrate (Millipore, USA).

### Apoptosis analysis

Cell apoptosis was determined by fluorescence-activated cell sorting (FACS) analysis as described [37].

### Co-Immunoprecipitation assay

Cells were harvested and total proteins were extracted as mentioned above. After determining protein concentrations, 500 μg of the total proteins lysate was brought to a final volume of 1 ml with RIPA. Crude cell extracts were precleared with Protein G Magnetic Beads (Millipore, USA) for 60 min at 4 °C. Indicated primary antibodies and IgG were added respectively into the lysates and incubated with constant rotation overnight at 4 °C. Protein G Magnetic Beads were then incubated to capture the added antibody for 120 min at room temperature. Captured immunoprecipitates were washed three times with PBS (add 0.1% tween-20) buffer and one time with diluted buffer. Then, the immunoprecipitates were suspended in SDS-PAGE loading buffer for immunoblot analysis.

### Statistical analyses

Data are expressed as means ± SD. The statistical significance of difference between groups was determined by student *t* test or one-way ANOVA. Correlations between SIRT3 and GSTP1 were evaluated using Spearman's σ rank test. All statistical analyses were performed using SPSS 19.0 software (IBM Corporation, USA).

## SUPPLEMENTARY MATERIALS FIGURES


