# Supplementary material for: Sirtuin 3 enhanced drug sensitivity of human hepatoma cells through glutathione S-transferase pi 1/JNK signaling pathway
Source: Oncotarget. 2016 Jun 29;7(31):50117–30. doi: 10.18632/oncotarget.10319 (PMC5226572; doi:10.18632/oncotarget.10319)
Supplement: Supplementary file 1 [file oncotarget-07-50117-s001.pdf]

Sirtuin 3 enhanced drug sensitivity of human hepatoma cells through glutathione S-transferase pi 1/JNK signaling pathway

SUPPLEMENTARY FIGURES

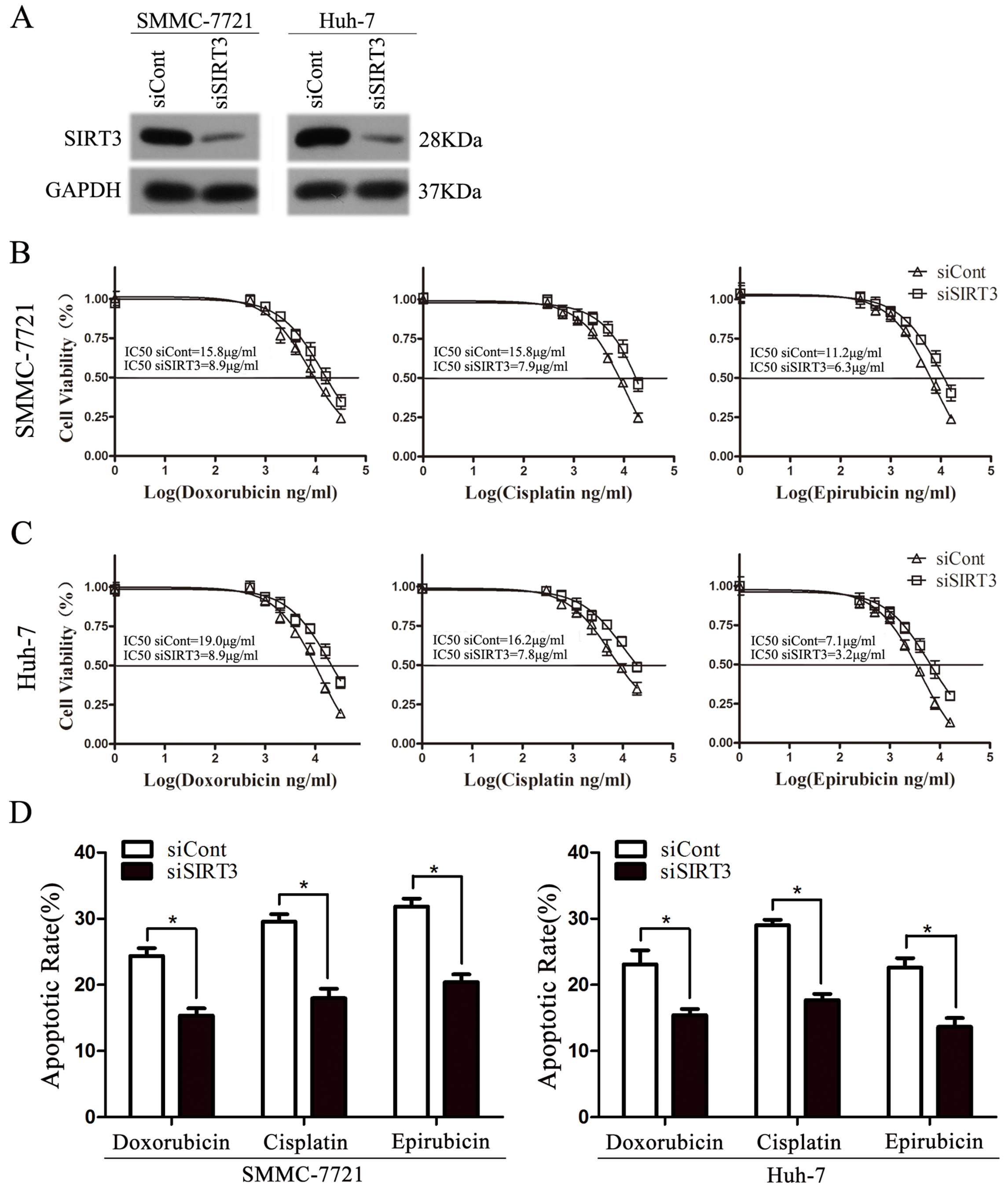

Supplementary Figure S1: SIRT3 knockdown in HCC cells induced drug resistance against chemotherapeutic agents. A. Western blotting analysis of SIRT3 proteins in cells transfected with siRNA targeting SIRT3 or control. GAPDH was used as a loading control. **B-C.** Cell viability of SIRT3-depleted cells exposed to chemotherapeutic agents. Forty-eight hour after siRNA transfection, SMMC-7721(B) and Huh-7(C) were treated with various concentrations of doxorubicin, cisplatin or epirubicin for 48 h. Cell viability was determined by MTS assay. **D.** Apoptosis in different groups was analyzed by flow cytometry with Annexin V/PI. Forty-eight hour after siRNA transfection, SMMC-7721 and Huh-7 cells were treated with doxorubicin (1 μg/ml), cisplatin (1 μg/ml) or epirubicin (0.5 μg/ml) for 48 h and subjected to flow cytometry analysis. *, *P*<0.05.

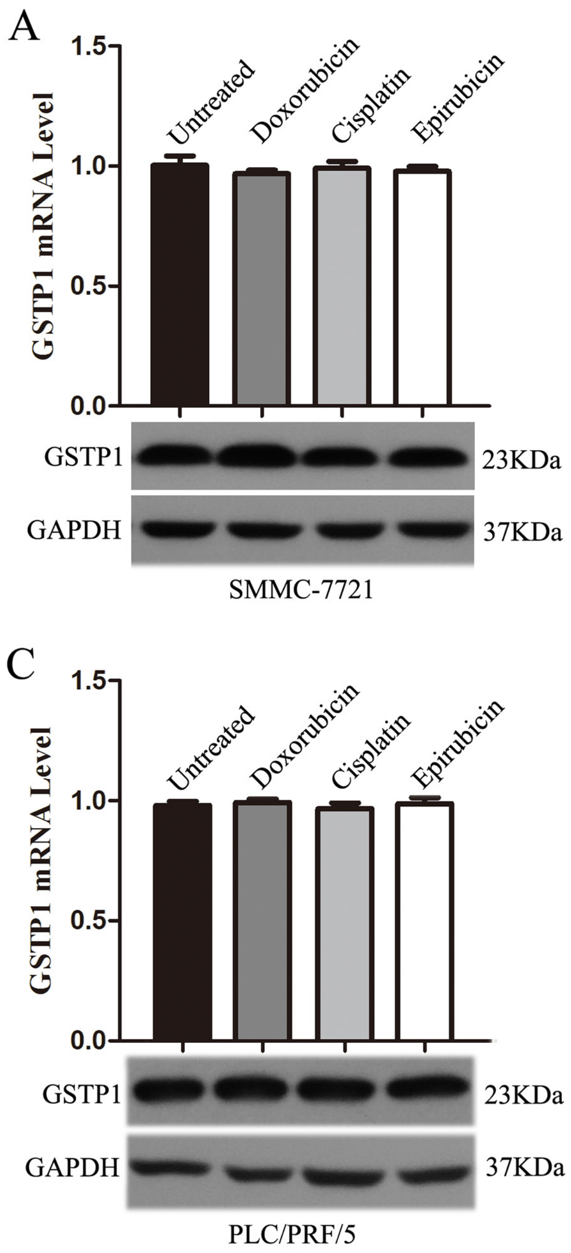

Supplementary Figure S2: The effect of chemotherapeutic agents on GSTP1 expression. A-C. SMMC-7721(A), Huh-7 (B) and PLC/PRF/5 (C) cells were treated with doxorubicin (1 µg/ml), cisplatin (1 µg/ml) or epirubicin (0.5 µg/ml) for 48 h and subjected to real-time PCR and western blotting analysis.

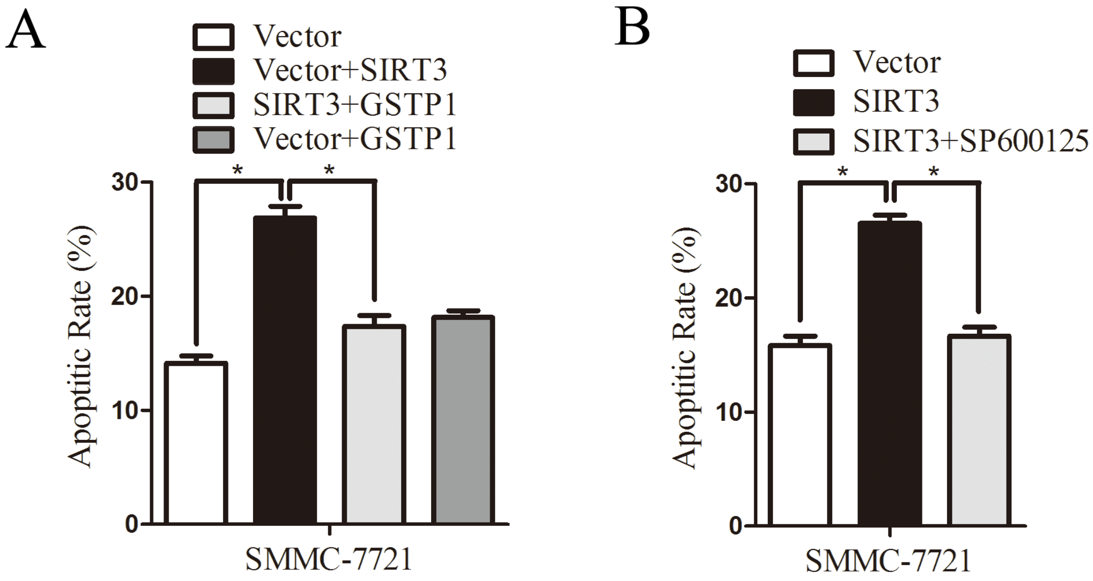

Supplementary Figure S3: GSTP1 overexpression or JNK inhibitor attenuate the sensitizing effect of SIRT3 to sorafenib. A. GSTP1 overexpression abolished SIRT3-induced apoptosis in HCC cells treated with sorafenib. SMMC-7721 stably expressing SIRT3 were transfected with plasmid expressing GSTP1 and were then exposed to sorafenib (2 µM) for 48 h. Apoptotic ratio in different groups was detected by flow cytometry with Annexin V/PI. *, $p<0.05$. **B.** JNK inhibitor abolished SIRT3-induced apoptosis in HCC cells treated with sorafenib. SMMC-7721 stably expressing SIRT3 were treated with JNK inhibitor SP600125 (10 µM) and sorafenib (2 µM). Apoptotic ratio in different groups was detected by flow cytometry. *, $p<0.05$.
